# Molecular detection of *Sodalis glossinidius*, *Spiroplasma* species and *Wolbachia* endosymbionts in wild population of tsetse flies collected in Cameroon, Chad and Nigeria

**DOI:** 10.1186/s12866-023-03005-6

**Published:** 2023-09-16

**Authors:** Youssouf Mouliom Mfopit, Judith Sophie Engel, Gloria Dada Chechet, Mahamat Alhadj Moussa Ibrahim, Djoukzoumka Signaboubo, Daniel Mbunkah Achukwi, Mohammed Mamman, Emmanuel Oluwadare Balogun, Mohammed Nasir Shuaibu, Junaidu Kabir, Soerge Kelm

**Affiliations:** 1 Institute of Agricultural Research for Development, Yaounde, Cameroon; 2 Africa Centre of Excellence for Neglected Tropical Diseases and Forensic Biotechnology, Ahmadu Bello University, Zaria, Nigeria; 3 Department of Biochemistry, Ahmadu Bello University, Zaria, Nigeria; 4 MARUM-PANGAEA, University of Bremen, Bremen, Germany; 5 Department of Public Health, Faculty of Human Health Sciences, University of N’Djamena, N’Djamena, Chad; 6 Institut de Recherche en Elevage pour le Développement, N’Djamena, Chad; 7 TOZARD Research Laboratory, P.O. Box 59, Bambili-Tubah, Bamenda, Cameroon; 8 Department of Veterinary Pharmacology and Toxicology, Faculty of Veterinary Medicine, Ahmadu Bello University, Zaria, Nigeria; 9 Department of Veterinary Public Health and Preventive Medicine, Ahmadu Bello University, Zaria, Nigeria; 10 Centre for Biomolecular Interactions Bremen, University of Bremen, Bremen, Germany

**Keywords:** Tsetse flies, Symbionts, *Wolbachia*, *Spiroplasma species*, *Sodalis glossinidius*, Cameroon, Chad, Nigeria

## Abstract

**Background:**

Tsetse flies are cyclical vectors of African trypanosomiasis (AT). The flies have established symbiotic associations with different bacteria that influence certain aspects of their physiology. Vector competence of tsetse flies for different trypanosome species is highly variable and is suggested to be affected by bacterial endosymbionts amongst other factors. Symbiotic interactions may provide an avenue for AT control. The current study provided prevalence of three tsetse symbionts in *Glossina* species from Cameroon, Chad and Nigeria.

**Results:**

Tsetse flies were collected and dissected from five different locations. DNA was extracted and polymerase chain reaction used to detect presence of *Sodalis glossinidius*, *Spiroplasma* species and *Wolbachia* endosymbionts, using species specific primers. A total of 848 tsetse samples were analysed: *Glossina morsitans submorsitans* (47.52%), *Glossina palpalis palpalis* (37.26%), *Glossina fuscipes fuscipes* (9.08%) and *Glossina tachinoides* (6.13%). Only 95 (11.20%) were infected with at least one of the three symbionts. Among infected flies, six (6.31%) had *Wolbachia* and *Spiroplasma* mixed infection. The overall symbiont prevalence was 0.88, 3.66 and 11.00% respectively, for *Sodalis glossinidius, Spiroplasma* species and *Wolbachia* endosymbionts. Prevalence varied between countries and tsetse fly species. Neither *Spiroplasma* species nor *S. glossinidius* were detected in samples from Cameroon and Nigeria respectively.

**Conclusion:**

The present study revealed, for the first time, presence of *Spiroplasma* species infections in tsetse fly populations in Chad and Nigeria. These findings provide useful information on repertoire of bacterial flora of tsetse flies and incite more investigations to understand their implication in the vector competence of tsetse flies.

**Supplementary Information:**

The online version contains supplementary material available at 10.1186/s12866-023-03005-6.

## Background

Trypanosomiasis disease is caused by trypanosomes, extracellular flagellated protozoan parasites of the genus *Trypanosoma*, and is one of major endemic diseases in sub-Saharan Africa. The disease exists as Human African Trypanosomiasis (HAT) (sleeping sickness) and the animal African trypanosomiasis (AAT) (nagana) forms. In humans, the disease is caused by *Trypanosoma brucei rhodesiense*, responsible for acute form of the disease in eastern and southern Africa, and *Trypanosoma brucei gambiense*, responsible for chronic form of the disease in western and central Africa [1]. Approximately 56 million people are estimated to be at different levels of risk of contracting HAT and more than 1.18 million Km^2^ are still at risk of *T. b. gambiense* infection [2]. In 2019, 992 cases recorded in Africa [1]. The AAT is caused by several species and subspecies of trypanosomes that include *Trypanosoma congolense*, *Trypanosoma vivax*, *Trypanosoma simiae*, *Trypanosoma uniforme*, *Trypanosoma godfreyi*, *Trypanosoma brucei brucei* and *Trypanosoma grayi*, and is a major constraint to Agricultural development on the continent, causing pathogenic infections in cattle, sheep, goats, pigs, dogs, camels and horses [3–5].

Trypanosomes are cyclically transmitted between different vertebrate hosts by tsetse flies (Diptera: Glossinidae). Thirty-one species and subspecies of tsetse flies have been described. The flies are grouped into three groups or subgenera based on common characteristics and morphology due to bio-ecological and genetic similarities; the riverine *Palpalis*, the savannah *Morsitans* and the forest *Fusca* [6, 7]. The flies acquire trypanosomes when feeding on an infected vertebrate host, which (trypanosomes) undergo a series of transformations and multiplication in their gut, giving rise to infective forms which inoculated into a new host during feeding [8].

Tsetse fly gut harbours diverse bacteria acquired from environment or maternally [9]. Previous studies have shown that these bacterial populations vary considerably depending both on tsetse fly species or sub-species and geographic origin of the flies [10]. The microbial community influences several aspects of tsetse fly’s physiology, including nutrition, fecundity development and maturation of innate immune system and vector competence [11, 12].

Tsetse flies have established long-term associations with four vertically transmitted endosymbiotic bacteria including *Wigglesworthia glossinidia*, *Sodalis glossinidius*, *Wolbachia species* and *Spiroplasma species* that was recently established as the fourth tsetse symbiont in *Glossina fuscipes fuscipes*, *Glossina tachinoides*, and *Glossina palpalis palpalis* [13]. They show different types of relation with their host.

All tsetse flies house *W. glossinidia* as primary and obligate endosymbiont. The *W. glossinidia* resides intracellularly within bacteriome in anterior midgut and extracellularly in milk gland of the fly [11]. The *W. glossinidia* provides dietary supplements absent in the tsetse fly vertebrate blood-meal restricted diet, supports larval development and contributes to maturation of adult immune system [12, 14]. *Wolbachia* endosymbionts are obligatory intracellular bacteria belonging to the Order Rickettsiales, infecting broad range of arthropod and filarial nematode species and probably most prevalent endosymbiont in insect germlines [11]. Within *Wolbachia* genus, 17 supergroups (A - Q) are currently recognized based on sequences of the five (*fbpA, coxA, ftsZ, gatB, coxA* and *hcpA*) conserved genes and the amino acid sequences of the four hypervariable regions of the *Wsp* protein [11, 15]. Majority of insect infections fall into supergroups A and B [16]. Phylogenetic analysis based on a concatenated dataset of MLST loci and *wsp* gene of *Wolbachia* strains detected in tsetse flies collected in ten African countries revealed that the *Wolbachia* strains infecting *G. m. morsitans*, *G. m. centralis*, *G. brevipalpis*, *G. pallidipes* and *G. austeni* belong to supergroup A, while the *Wolbachia* strain infecting *G. p. gambiensis* fell into supergroup B [17].

In tsetse fly, *Wolbachia* mainly resides in reproductive tissues and is maternally transmitted from generation to generation through trans-ovarian transmission. *Wolbachia* is also transferred horizontally among arthropods. *Wolbachia* infection in the tsetse fly host results in a variety of reproductive abnormalities such as parthenogenesis, male killing, feminization and cytoplasmic incompatibility [16–18]. Cytoplasmic incompatibility (CI) results in embryonic mortality in progeny derived from mating between insects with different *Wolbachia* infection status, where infected male mates with an uninfected female or infected female with a different strain of the bacterium [16]. The presence of this symbiont in the *Glossina morsitans morsitans* tsetse fly is associated with the induction of CI [18], which confers indirect reproductive advantages to infected females and is considered as a potential vector control alternative [11, 18]. Some studies have shown that *Wolbachia* infections limit mosquito-transmitted pathogens including dengue virus, chikungunya virus, *Plasmodium* parasites, yellow fever virus, Zika virus and filarial nematodes [19–22].

The tsetse fly’s secondary and facultative symbiont is the commensal *S. glossinidius*. It is a gram-negative organism belonging to Enterobacteriaceae family. The *S. glossinidius* is widely spread in numerous tissues of tsetse fly (midgut, fat body, milk gland, salivary glands and reproductive system), present intracellularly and extracellularly [23]. The *S. glossinidius* genome consists of one circular chromosome of 4.17 Mbp, three extrachromosomal plasmids designated pSG1, pSG2, and pSG3, as well as a phage, ФSG1. However, its genome sequence shows reduced coding capacity with a large number of pseudogenes [24]. The *S. glossinidius* can be transmitted maternally via haemolymph, milk gland secretions, and horizontally during mating [11, 25]. In tsetse flies, specific role of this symbiont is still not clear. However, it has been shown to affect host longevity and has been suspected to play a role in potentiating susceptibility to trypanosome infection in tsetse by influencing efficacy of tsetse immune system possibly through lectin-inhibitory activity [26].

The *Spiroplasma* genus belongs to Mollicutes class, and Tenericutes phylum. *Spiroplasma* species are abundant in insects gut or haemolymph where they have large variety of commensal, pathogenic or mutualist interactions with host [13]. *Spiroplasma* confers protection against pathogens such as fruit fly *Drosophila neotestacea* from nematode [27], pea aphid *Acyrthosiphon pisum* against fungi [28] and fruit fly *Drosophila hydei* against a parasitoid wasp *Leptopilina heterotoma* [29]. However, reproductive alterations such as CI, male-killing and sex determination are related to numerous species of *Spiroplasma* [13].*Spiroplasma* recently, been established as a new class of tsetse fly symbiont in *G. f. fuscipes*, *G. tachinoides*, and *G. p. palpalis*. The interactions between *Spiroplasma* and tsetse flies seem to be beneficial because of its ability to extend lifespan and reduce t vector competence for *Trypanosoma* [11, 30].

Current control measures against trypanosomiasis are mainly based on chemotherapy. In absence of effective vaccine and to address limitations associated with chemotherapy, disruption of trypanosomes transmission through vector control is crucial. Transmission of pathogens by vector depends on its vector competence, which can be affected by several factors, including vector endosymbionts [31]. Due to their importance, interactions between the symbionts and their hosts are being harnessed toward development of novel approaches for vector and disease control [32–34].

The present study assessed presence of *Sodalis glossinidius*, *Spiroplasma* species and *Wolbachia* in wild populations of tsetse flies in Cameroon, Nigeria and Chad.

## Results

For this study, 848 tsetse fly samples from Cameroon, Chad and Nigeria were used. The flies belonged *Glossina morsitans submorsitans*: 403 (47.52%); *G. p. palpalis*: 316 (37.26%); *G. f. fuscipes*: 77 (9.08%); *G. tachinoides*: 52 (6.13%) species. Distributions of prevalence of different symbiotic bacteria are presented in Table 1.

**Table 1:** Distributions of endosymbiont infection in respective study areas

Countries	Sites	Species	*S. glossinidius*	*Spiroplasma*	*Wolbachia*
**Cameroon**	Dodeo	*G. p. palpalis*	3/149 (2.01%)	0/154 (0.0%)	15/155 (9.68%)
			**3/149 (2.01%)**	**0/154 (0.0%)**	**15/155 (9.68%)**
**Chad**	Maro	*G. f. fuscipes*	2/69 (2.90%)	2/69 (2.90%)	8/69 (11.59%)
	Lac Iro	*G. f. fuscipes*	0/6 (0.0%)	0/8 (0.0%)	0/2 (0.0%)
		*G. m. submorsitans*	1/182 (0.55%)	2/343 (0.58%)	3/86 (3.49%)
		*G. tachinoides*	0/2 (0.0%)	0/3 (0.0%)	
			**3/259 (1.16%)**	**4/423 (0.95%)**	**11/157 (7.00%)**
**Nigeria**	Ija-Gwari	*G. p. palpalis*	0/161 (0.0%)	20/161 (1.24%)	23/161 (14.29%)
	Yankari	*G. m. submorsitans*	0/60 (0.0%)	4/60 (6.66%)	4/60 (6.67%)
		*G. tachinoides*	0/49 (0.0%)	3/49 (6.12%)	11/49 (22.45%)
			**0/270 (0.0%)**	**27/270 (10.0%)**	**38/270 (14.07%)**
		**Total**	**6/678 (0.88%)**	**31/847 (3.66%)**	**64/582 (11.00%)**

Out of 848 flies, 95 (11.20%) were infected with at least one of the endosymbionts. Among infected flies, six (6.31%) had *Wolbachia*-*Spiroplasma* mixed infection.

### *Sodalis glossinidius* infection prevalence

**Fig. 1:**
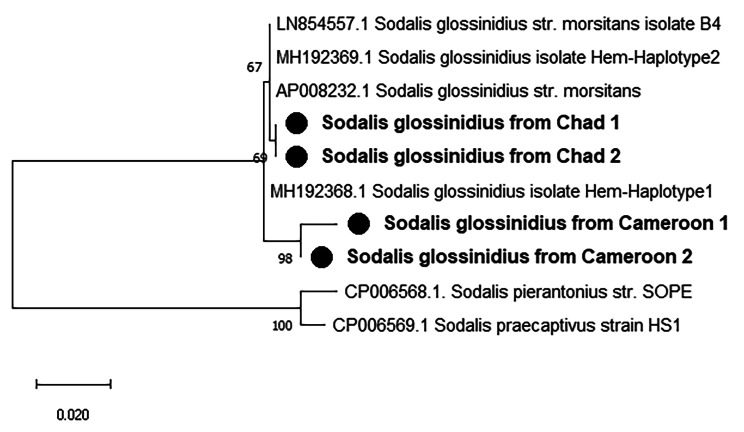
Phylogenetic tree of detected. *Sodalis glossinidius’ hemolysin* partial gene and its closed relatives. The evolutionary history conducted in MEGA X, was inferred by using the Maximum Likelihood method and Hasegawa-Kishino-Yano model. This analysis involved 10 nucleotide sequences and a total of 596 positions in the final dataset. Our isolates are marked by a black circle. The percentage of replicate trees in which the associated taxa clustered together in the bootstrap test (1000 replicates) are shown next to the branches

Presence of *S. glossinidius* was investigated in 678 field-collected tsetse flies from Cameroon (149), Chad (259) and Nigeria (270) using a *Sodalis Hemolysin* gene-based PCR. The *S. glossinidius* infection rates were 2.01%, 1.16% and 0.0% in Cameroon Chad Nigeria respectively (Table 1). Prevalence of *S. glossinidius* infection were similar (p > 0.05) between Cameroon and Chad. The sequence of our amplicons had > 99% similarity to *Sodalis’s Hemolysin* partial gene sequences (OQ458712.1, MH192369.1, LN854557.1, AP008232.1) in NCBI database. Maximum Likelihood phylogenetic tree (Fig. 1) revealed amplicons from Cameroon and Chad with different clades.

### *Spiroplasma* infection prevalence

A total of 847 fly samples from Cameroon (154), Chad (423) and Nigeria (270) were used for molecular detection of *Spiroplasma* using a 16 S rRNA-based PCR approach with wspecF/wspecR primers. *Spiroplasma* was detected in 31 (3.66%) of the samples, with. prevalence varying significantly (p < 0.001) between countries (Table 1). *Spiroplasma* was detected in 0.0%, 0.95% and 10.00% samples from Cameroon, Chad and Nigeria respectively. The sequence of our amplicons had > 99% similarity to *Spiroplasma* 16 S rRNA partial gene sequences (KX159383.1, NR 104720.1, OQ448933.1, CP018022.1) in NCBI database.

**Fig. 2:**
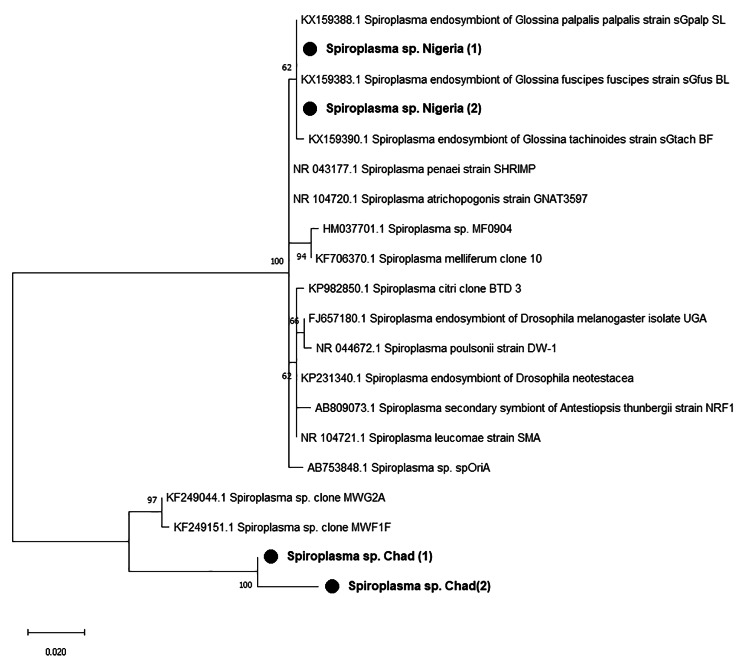
Phylogenetic tree of detected *Spiroplasma’s* 16S rRNA partial gene and its closed relatives. The evolutionary history conducted in MEGA X, was inferred by using the Maximum Likelihood method and Kimura 2-parameter model. This analysis involved 20 nucleotide sequences and a total of 383 positions in the final dataset. Our isolates are marked by a black circle. The percentage of replicate trees in which the associated taxa clustered together in the bootstrap test (1000 replicates) are shown next to the branches

Maximum Likelihood phylogenetic tree (Fig. 2) revealed amplicons from Nigeria and Chad with different clades.

### *Wolbachia* infection prevalence

**Fig. 3:**
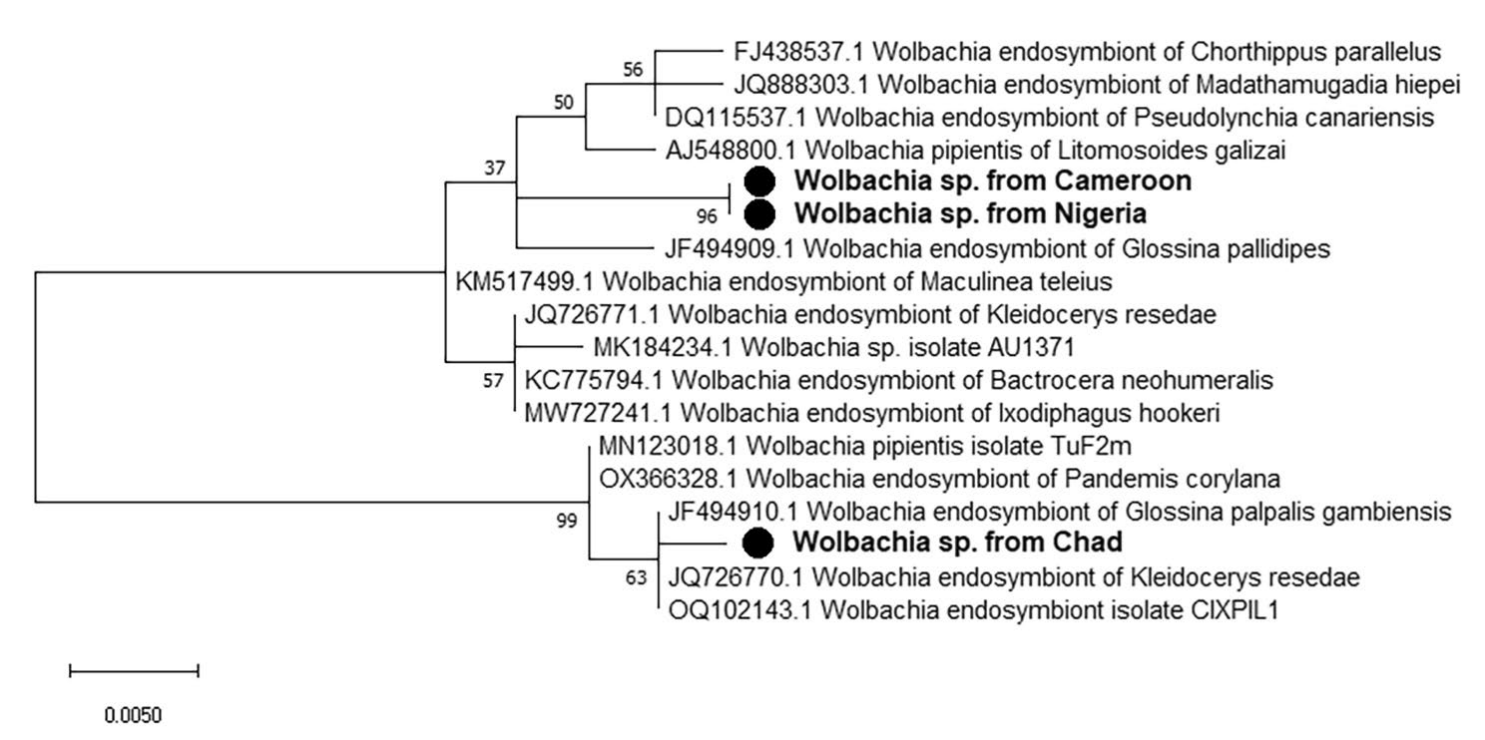
Phylogenetic tree of detected *Wolbachia’s* partial 16 S rRNA gene and its closed relatives. The evolutionary history conducted in MEGA X, was inferred by using the Maximum Likelihood method and Kimura 2-parameter model. A discrete Gamma distribution was used to model evolutionary rate differences among sites. This analysis involved 18 nucleotide sequences and a total of 377 positions in the final dataset. Our isolates are marked by a black circle. The percentage of replicate trees in which the associated taxa clustered together in the bootstrap test (1000 replicates) are shown next to the branches

A total of 582 tsetse samples (gut or abdomen) were screened for presence of *Wolbachia* using 16 S rRNA-based PCR approach with wspecF/wspecR set of primers. The samples were collected in Cameroon: 155, Chad: 157 and Nigeria: 270. The results of the screening (Table 1) revealed that prevalence of *Wolbachia* infections varied, but not significantly (p = 0.06) between the three countries. The *Wolbachia* prevalence in Cameroon, Chad and Nigeria was 9.68%, 7.00% and 14.07% respectively. The sequence of our amplicons had > 99% similarity to *Wolbachia* 16 S rRNA gene sequences (MK277440.1, JQ726770.1, JF494910.1, KM517499.1) in NCBI database. Maximum Likelihood phylogenetic tree (Fig. 3) revealed amplicons from Nigeria and Cameroon on same clades, and separate from that of chad.

All 16 S rRNA and hemolysin gene sequences generated in this study are deposited into GenBank under accession numbers OQ448931 to OQ448937 and OQ458709 to OQ458712 (See additional file 1).

### Prevalence according to the fly species

All the four tsetse fly species were infected at different rates by all the endosymbionts, except *G. tachinoides* flies that was devoid of *S. glossinidius* infections (Table 2). There was no significant difference (p = 0.28) between the prevalence of *S. glossinidius* among the four species. The prevalence of *Spiroplasma* was significantly higher (p = 0.005) in *G. p. palpalis* (6.35%) and *G. tachinoides* (5.77%) compared to *G. m. submorsitans* (1.49) and *G. f. fuscipes* (2.60%). For *Wolbachia*, the prevalence was significantly lower (p = 0.005) in *G. m. submorsitans* (4.79%) compared to other fly species.

**Table 2:** Distribution of endosymbiont infection per tsetse fly species

	*S. glossinidius*	*Spiroplasma*	*Wolbachia*
***G. f. fuscipes***	2/75 (2.67%)	2/77 (2.60%)	8/71 (11.27%)
***G. m. submorsitans***	1/242 (0.41%)	6/403 (1.49%)	7/146 (4.79%)
***G. p. palpalis***	3/310 (0.97%)	20/315 (6.35%)	38/316 (12.02%)
***G. tachinoides***	0/51 (0.0%)	3/52 (5.77%)	11/49 (22.45%)
p-value (Pearson χ^2^)	**0.283**	**0.005**	**0.005**

When the collection site is considered (prevalence compared between different fly species living in the same environment), symbiont prevalence among the three tsetse species in Lac Iro were similar. However, in Yankari collection site, the *Wolbachia* infection was significantly higher (p = 0.017) in *G. tachinoides*.

## Discussion

In this study, tsetse symbiotic bacteria (*S. glossinidius*, *Spiroplasma* and *Wolbachia*) were detected in 11.20% of tsetse samples examined with varying prevalence within countries, collection sites and tsetse species. This overall symbiont infection rate is lower compared to that of other previous studies [35–37]. Varying levels of infection rates may be attributed to environmental factors (vegetation, humidity, temperature) encountered in different ecological settings and to the intrinsic characters of each tsetse species.

The overall *S. glossinidius* infection rate of 0.88% obtained in the present study is much lower than the 12.69% global prevalence of *Sodalis* in tsetse samples from 15 African countries [35]. However, in the same study, similar prevalence was obtained in Burkina Faso, Mali, Ghana and Senegal. The *Sodalis* prevalence in Chad (1.16%) was lower than the 9.0% previously reported in the same area [38]. The prevalence in Cameroon was also lower than the 37.2% previously reported in the neighbouring area [36]. The variation may be due to PCR screening approach. The primers used in the previous works (pSG2 primers) were targeting a portion of the extrachromosomal plasmid (pSG2) while in this present work, the primers (Hem primers) were targeting the nuclear hemolysin gene. Comparing different sets of primers, it has been reported that the use of the hemolysin gene provided a more reliable assessment of prevalence than the pSG2 that was giving higher but non-consistent prevalence [39]. Moreover, tsetse flies screened in Cameroon for in the present study were all *G. p. palpalis* while the previous study screened *G. tachinoides* and *G. m. submorsitans*. There is no published data on *S. glossinidius* prevalence in tsetse flies in Nigeria. The presence of *S. glossinidius* has been suspected to be involved in vector competence of tsetse fly. But the confirmation is still under debate. Numerous reports showed a positive association between *Sodalis* and trypanosome establishment in the midgut possibly through lectin-inhibitory activity involving the production of N-acetyl glucosamine [26, 38, 40]. However, other studies reported absence of association between the presence of *Sodalis* and that of Trypanosome [35–37].

The *Spiroplasma* general infection rate of 3.66% obtained in present study is much lower than 17.17% and 44.5% reported respectively in Burkina Faso for *G. tachinoides* and in Uganda for *G. f. fuscipes* [30, 41]. The study in Uganda showed a high (0–62% variation of prevalence across the 26 sampling sites ranging and was correlated with geographic origin and season of collection of *G. f. fuscipes* [30]. The prevalence in Nigeria (10.0%) was significantly higher (p = 0.00) compared to that of Cameroon (0.0%) and Chad (= 0.95%). Few investigations have been done on the prevalence of *Spiroplasma* in tsetse flies in Africa. There is no data on tsetse *Spiroplasma* prevalence in the three countries where our samples were collected. *Spiroplasma* is known to induce reproductive abnormalities and pathogen protective phenotypes in various arthropods hosts [11]. A negative correlation of *Spiroplasma* with trypanosome infection found in *G. f. fuscipes*, indicate that *Spiroplasma* infections may have an important effect in tsetse fly resistance to infection with trypanosomes [30, 42]. Experimental study by Son et al. [42] on *G. f. fuscipes* established that *Spiroplasma* infection induced changes in sex–biased gene expression in the reproductive tissues, a depletion in the availability of nutrients in pregnant females resulting in delayed larval development, and compromised sperm fitness. These findings indicate that *Spiroplasma* could be exploited for reducing tsetse population size and therefore, the disease transmission [42].

The *Wolbachia* global prevalence of 11.00% and countries prevalence obtained in the present study are in the range of prevalence reported by Doudoumis et al. [17]. All these values were lower than 80.5% and 78.9% reported in Zambia for *G. m. morsitans* and *G. pallidipes* respectively [37]. In Cameroon and Chad, the prevalence previously obtained in the same and neighbouring study sites (67.6% and 14.5% respectively) were much higher than those of the present study [36, 38]. The primers used in this study were targeting the *Wolbachia* specific 16 S rRNA while the previous studies used primers targeting the *Wolbachia* surface protein (*wsp*) gene. The comparison of results generated by 16 S rRNA and *wsp* primers by [43] did not find significant difference between the two primers, even though the prevalence with 16 S rRNA primers (65%) was higher than the prevalence with wsp primers (60.5%). The tsetse species were globally different amongst the two studies. The prevalence in Nigeria (14.07%) was relatively higher than those of the two other countries (Cameroon: 9.68% and Chad: 7.00%). There is no data on *Wolbachia* infection in tsetse flies in Nigeria.

*Wolbachia* has received increased interest in recent years because of its high rates of distribution in a wide range of arthropods and nematodes, its unique effects on host physiology and its potential in disease control. Concerning trypanosomiasis, there is no confirmed implication of *Wolbachia* in tsetse vector competence. Investigations on the tripartite association between tsetse fly, *Wolbachia* and trypanosomes reported contrasting results. When in *G. f. fuscipes* there was a negative association between *Wolbachia* and trypanosome [44] suggesting prevention of trypanosome infections by the presence of *Wolbachia*; some studies did not find association between presence of *Wolbachia* and ability of tsetse flies to harbor trypanosomes [36, 37, 43].

*Spiroplasma* infected the four tsetse fly species, but with a low infection rate on *G. m. submorsitans* and *G. f. fuscipes* compared to *G. tachinoides* and *G. p. palpalis*. On the contrary, the investigations by Doudoumis et al. [13] found *Spiroplasma* infection only in palpalis group and none in the morsitans and fusca groups. Attributing this result to their frequent infection with *Wolbachia* which may have led to development of competitive exclusion with *Spiroplasma*. *Wolbachia* was detected in all the four tsetse fly species with varying prevalence within species. This variation agrees with findings from similar studies on tsetse fly symbionts. In the present study, *G. tachinoides* was found to be more infected with *Wolbachia* than the three other tsetse fly species. The *S. glossinidius* was not detected in *G. tachinoides*, but has been detected in *G. tachinoides* in other studies [35, 36].

## Conclusion

The present study revealed for the first time, the presence of infection by *Spiroplasma* in tsetse flies in Chad and Nigeria. The infection rates of *S. glossinidius*, *Spiroplasma* species and *Wolbachia* varied between countries and collection sites probably due to environmental factors. Few tsetse flies harboured symbiont co-infections. These findings provide useful information to repertoire of bacterial flora of tsetse flies and call for more investigations to understand the implication of these symbiotic bacteria in the vector competence of tsetse flies. More data could help development of environmentally friendly methods for tsetse and trypanosomiasis control.

## Methods

### Study areas

**Fig. 4:**
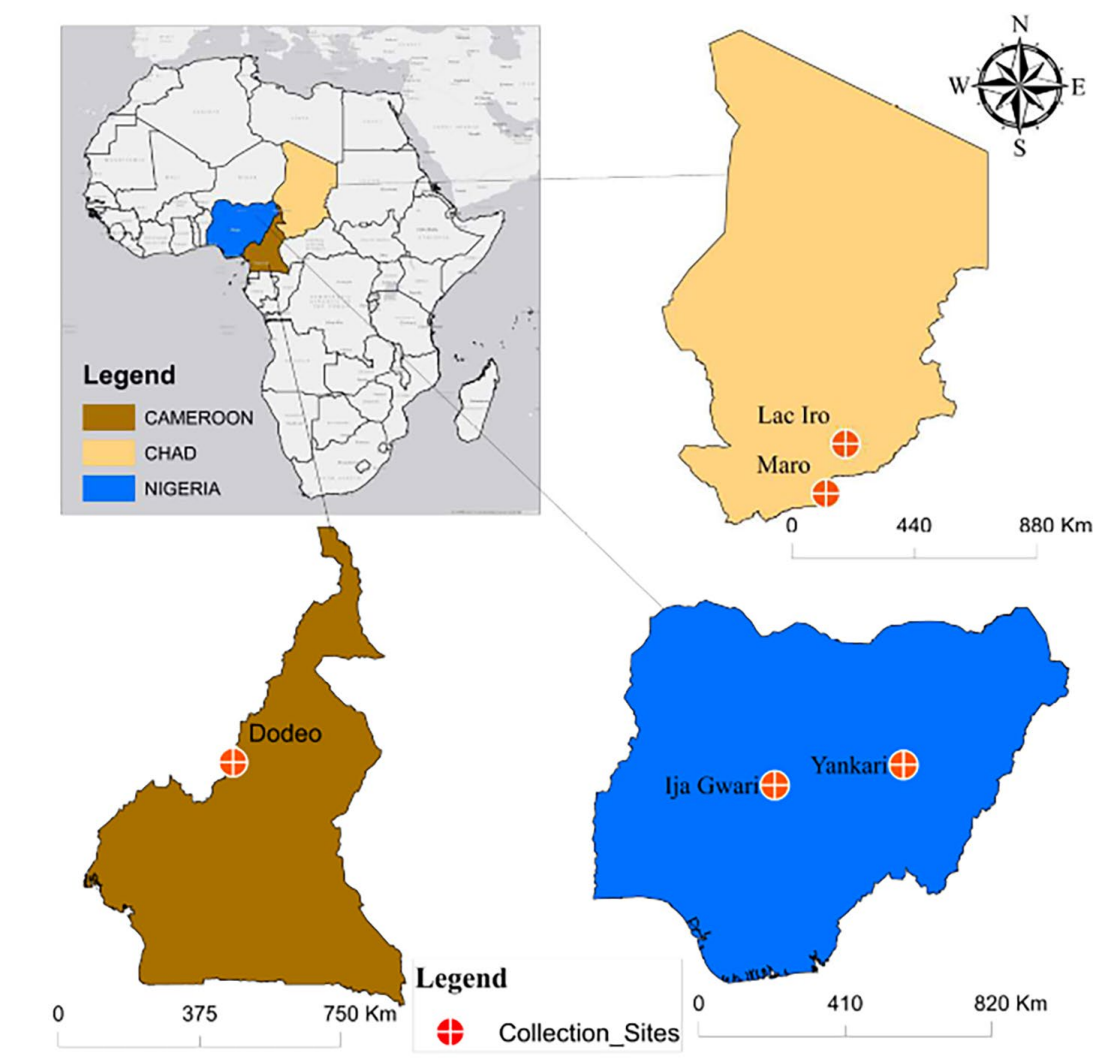
Study area. Tsetse flies were collected in Cameroon (Dodeo), in Chad (Maro and Lake Iro) and in Nigeria (Yankari Game reserve and in Ija-Gwari).

Tsetse flies used in this study were collected from Cameroon, Chad, Nigeria (Fig. 4). In Cameroon flies were trapped in Dodeo (Latitude 7° 27.994’ N and Longitude 12° 04.101′ E) located in the “Faro et Déo” division of the Adamawa region. The village is close to the Cameroon-Nigeria Border. The vegetation is predominantly characterized by gallery forest along rivers and the climate type is of Sudano-Sahelian with rainy season from May to October and a dry season from November to April. This area has a dense hydrographic network and offers pastures for livestock [5, 36].

In Chad, flies were collected in Maro and Lake Iro areas, all situated in the Moyen-Chari province in southern Chad. The area has a climate of Sudano-Sahelian type with A rainy season from May to October and a dry season from November to April of equal duration. Lac Iro is situated between Latitude 9°59’ N and Longitude 19°26′ E, along Salamat River. The vegetation is dominated by floodplains and dense forests containing shrubs. The area is considered as a buffer zone of Zakouma National Park where domestic and wild animals meet [38]. Maro (8°24.807’ N and 18°46.139’ E) is located at Chad-Central African Republic border. The area is crossed by many rivers and their multiple tributaries; the most important is the Chari River and its confluent the Grande Sido [45]. Maro is close to the Bamingui-Bangouran National Parc.

In Nigeria, flies were collected in Yankari and in Ija-Gwari. Yankari Game Reserve (9°45.240’N and 10°30.448E) is situated in the south-central part of Bauchi State in the North-East of the country. It covers an area of 2244 km^2^ and has a Sudan savannah vegetation. It has well-developed patches of woodland with scattered shrubs and trees. This national park has a rich wildlife diversity. Ija-Gwari is located at 9° 18.860’N and 7° 26.814’E in Tafa Local Government Area of Niger State. The vegetation is of riverine fringing forest forming a dense two-storey canopy. The collection site is an open area where human activities (agriculture and cattle grazing) are regular [46].

### Sampling of tsetse

Biconical traps, very effective in catching riverine tsetse of the palpalis group [47] and widely used throughout Africa were used for this study, set up in various tsetse fly-favourable biotopes at least 100 m intervals. Tsetse flies were collected once a day and transported in cool boxes to a base camp. The collected flies were identified using morphological identification keys [48, 49], numbered and dissected on the same day.

### Dissection

Flies were dissected in a drop of sterile saline solution as described by Ngomtcho et al. and Shaida et al., [5, 46]. The wings were removed first, followed by legs, proboscis, salivary glands and gut from each fly. After each fly, dissection tools (forceps, pins and slide) were decontaminated by immersion in 5% sodium hypochlorite for approximately ten minutes, followed by immersion in 70% ethanol and final immersion in sterile normal saline. Wings were stored dry for geometric morphometrics. Legs, proboscis and salivary glands were preserved in nucleic acid preservation agent (NAPA: 25 mM sodium citrate, 10 mM EDTA, 70 g ammonium sulphate/100 mL solution, pH 7.5) in 1.5 mL cryotubes. Gut tissue was homogenised in 200 µL of 50 mM Tris-Cl, pH 9.0, using four 2.38 mm metal beads (MoBio Laboratories, Carlsbad, CA, USA). Fifty microliters of the homogenate were added to 500 µL of NAPA. Most of the flies from Chad were dead. For this reason, after the removal of wings, legs and proboscis, the entire abdomen was transferred in ethanol.

### DNA extraction

DNA was extracted from homogenised gut tissue in NAPA with the DNeasy Blood and Tissue Kit (Qiagen, Germany) according to the manufacturer’s instructions with slight adjustments (100 µL of homogenate were used for purification and 100 µL elution buffer were used at elution step).

For the dead flies from Chad, DNA was extracted from the abdomen using 5% Chelex-100 Resin (BIO-RAD, Hercules, California, USA). Briefly, the abdomen was transferred from ethanol to a new 1.5 mL centrifuge tube and allowed to dry. The abdomen was then crushed using the micro-pipette tips. Thereafter, 100 µL of chelex 5% solution were added. The mixture was vortexed and incubated at 56° C for 30 min in a Thermomixer. After this incubation, the mixture was vortexed and incubated for an additional 5 min at 95° C. The mixture was then mixed thoroughly before brief centrifugation at 7000 rpm for 1 min and stored at -20 °C.

### Molecular detection of *Wolbachia*

The detection of *Wolbachia* was carried out by amplifying a 438 bp fragment of the 16 S rRNA gene with the 16 S W-Spec primers (Table 3) designed by Werren & Windsor [50]. PCR amplifications were performed in 20 µL reaction mixture containing 1 X DreamTaq buffer (10X), 150 µM dNTPs, 0.2 µM of each primer, 0.5 U of DreamTaq polymerase and 2 µl of template DNA. The cycling condition was as described by Doudoumis et al. [17]. It started by 95 °C for 5 min followed by 35 cycles of 95 °C for 30 s, 30 s at 54 °C, 1 min at 72 °C and a final extension step of 72 °C for 10 min. *Wolbachia* positive control sample was the genomic DNA from *Glossina morsitans morsitans* infected with *Wolbachia*, donated by the laboratory of Alvaro Acosta Serrano at the Liverpool School of Tropical Medicine. For negative control, ddH_2_O was used instead of fly DNA template. After amplification, the PCR products were analyzed by electrophoresis on a 1.5% agarose gel containing Stain-G and visualized under UV light. PCR products of one *Wolbachia* positive sample was randomly selected in each country and sequenced.

**Table 3:** List of primers used in this study

Organisms	Primer name	Nucleotide sequences (5’ – 3’)	Amplicon size (bp)	References
*S. glossinidius*	HemF	ATGGGAAACAAACCATTAGCCA	650	[51]
	HemR	TCAAGTGACAAACAGATAAATC		
*Spiroplasma*	63 F	GCCTAATACATGCAAGTCGAAC	455	[13]
	TKSSsp	TAGCCGTGGCTTTCTGGTAA		
*Wolbachia*	WspecF	CATACCTATTCGAAGGGATAG	438	[50]
	WspecR	AGCTTCGAGTGAAACCAATTC		

### Molecular detection of *S. glossinidius*

The presence of *S. glossinidius* was determined by PCR with Hem primers (Table 3) that target the gene encoding the haemolysin protein of the bacterium [51]. The primers targeted a 650 bp fragment of the hemolysin gene. The PCR reaction was performed in a final volume of 20 µL containing 1X Dream taq buffer, 150 µM dNTPs, 0.2 µM of each primer, 0.5 U of Dream Taq polymerase and 2 µL of DNA template. PCR cycles were: 95 °C for 5 min; 35 cycles of 95 °C for 30 s, 54 °C for 30 s and 72 °C for 60 s; and a final elongation at 72 °C for 10 min. *S. glossinidius* positive control sample was the genomic DNA from *Glossina morsitans morsitans* infected with *S. glossinidius*, donated by the laboratory of Alvaro Acosta Serrano at the Liverpool School of Tropical Medicine. For negative control, ddH_2_O was used instead of fly DNA template.

After amplification, the PCR products were analyzed by electrophoresis on a 1.5% agarose gel containing Stain-G and visualized under UV light. PCR products of two *S. glossinidius* positive samples were randomly selected in each country and sequenced.

### Molecular detection of *Spiroplasma*

Screening for *Spiroplasma* was carried out by amplifying 455 bp fragment of 16 S rRNA as described by Doudoumis et al. [13] with specific primers (Table 3). PCR reactions were performed in 20 µl reaction mixture containing 1X DreamTaq buffer, 150 µM dNTPs, 0.65 µM of each primer, 0.5 U of DreamTaq polymerase and 2 µl of template DNA. The PCR temperature profile was 95 °C for 5 min followed by 35 cycles of 95 °C for 30 s, 30 s at 59 °C, 1 min at 72 °C and a final extension step of 72 °C for 10 min.

To obtain *Spiroplasma* positive control, a PCR with randomly selected samples was ran. The sample with the band at the target position was re-amplified and its PCR product was purified and sequenced for confirmation. For Negative controls, ddH_2_O was used instead of the DNA template.

After amplification, the PCR products were analyzed by electrophoresis on a 1.5% agarose gel containing Stain-G and visualized under UV light. PCR products of two *Spiroplasma* positive samples were randomly selected in each country and sequenced.

### Sequencing

Selected positive samples, were re-amplified in PCR reaction volume of 50 µL (maintaining the same concentration of reactants). Five microliters were visualised on 1.5% agarose gel for confirmation of the amplification. The remaining 45 µL PCR products were separated on 2% agarose gel. The amplicons bands were extracted and purified using GeneJET Gel Extraction Kit (Thermo Scientific) according to manufacturer instructions. The purified amplicons were sent for sanger sequencing to a commercial company (SeqLab, Göttingen, Germany).

### Phylogenetic analysis

Obtained sequences were manually checked and edited using Geneious Pro version 5.5.9 software. The Basic Local Alignment Search Tool (BLASTn) [52] from National Center for Biotechnology Information (NCBI) was used to confirmed identity of amplicons and to determine closest related sequences in the GenBank. Sample sequences and reference sequences obtained from NCBI were aligned using MUSCLE [53] alignment tool with its default setting implemented in MEGAX [54] which was also used to infer phylogenetic relationships. Maximum likelihood method was performed with the Kimura-2 model [55] for *Wolbachia* and *Spiroplasma*, then Hasegawa-Kishino-Yano model [56] for *Sodalis* as determined by the MEGA model finder tool with 1000 bootstraps replicates.

### Statistics data analysis

Endosymbionts hosted by tsetse flies were expressed in percentage as symbiont prevalence. The Pearson’s chi-square test (χ^2^) was used to compare symbiont prevalence between countries and collection sites. The differences were considered significant when the p-values were lower than 0.05. Statistical tests were performed using SPSS for Windows, version 20.

### Electronic supplementary material

Below is the link to the electronic supplementary material.

**Additional file 1:** Sequence accession numbers. Table indicating which accession number corresponds to each sequence.

## Data Availability

The datasets generated and analysed during this study are included in this publication. The 16 S rRNA and hemolysin partial gene sequences generated have been deposited into GenBank under accession numbers OQ448931 to OQ448937 and OQ458709 to OQ458712.

## List of abbreviations

BLAST: Basic Local Alignment Search Tool
NAPA: Nucleic Acid Preservation Agent
NCBI: National Center for Biotechnology Information
