# Supplementary material for: Molecular detection of Sodalis glossinidius, Spiroplasma species and Wolbachia endosymbionts in wild population of tsetse flies collected in Cameroon, Chad and Nigeria
Source: BMC Microbiol. 2023 Sep 16;23:260. doi: 10.1186/s12866-023-03005-6 (PMC10504758; doi:10.1186/s12866-023-03005-6)
Supplement: Supplementary file 1 — Additional file 1: Sequence accession numbers. Table indicating which accession number corresponds to each sequence. [file 12866_2023_3005_MOESM1_ESM.docx]

**Additional file 1: Sequence accession numbers**

| **Accession N°** | **Organism/gene** | **Location** | **Sample** |
| --- | --- | --- | --- |
| OQ448931 | *Spiroplasma* 16S rRNA | Chad | CHD198 |
| OQ448932 | *Spiroplasma* 16S rRNA | Chad | CHD9 |
| OQ448933 | *Spiroplasma* 16S rRNA | Nigeria | NGR60 |
| OQ448934 | *Spiroplasma* 16S rRNA | Nigeria | NGR61 |
| OQ448935 | *Wolbachia* 16S rRNA | Cameroon | CMR1260 |
| OQ448936 | *Wolbachia* 16S rRNA | Chad | CHD263 |
| OQ448937 | *Wolbachia* 16S rRNA | Nigeria | NGR87 |
| OQ458709 | *S. glossinidius* Hemolysin | Cameroon | CMR1232 |
| OQ458710 | *S. glossinidius* Hemolysin | Cameroon | CMR1263 |
| OQ458711 | *S. glossinidius* Hemolysin | Chad | CHD279 |
| OQ458712 | *S. glossinidius* Hemolysin | Chad | CHD388 |
